# Effects of curcumin supplementation on homocysteine levels: a systematic review and meta-analysis of randomized controlled trials

**DOI:** 10.3389/fphar.2026.1864151

**Published:** 2026-07-16

**Authors:** Hong Cao, Wenrui Ning, Feng Zhang, Xin Zhang, Jiai Yan, Yingyu Wang, Jing Sun, Yiran Liu, Dan Li, Ju Yang

**Affiliations:** 1 Nutrition Department (Center for Clinical Evaluation of Functional Foods), Affiliated Hospital of Jiangnan University, Wuxi, China; 2 School of Biotechnology, Jiangnan University, Wuxi, China; 3 Wuxi Medical College, Jiangnan University, Wuxi, China; 4 Jiangsu Jicui Future Food Technology Research Institute Co., Ltd., Yixing, China; 5 Endocrine Department, Jiangnan University Affiliated Hospital, Wuxi, China

**Keywords:** curcumin, homocysteine, meta-analysis, randomized controlled trial, cardiovascular risk

## Abstract

**Background:**

Homocysteine (Hcy) is an established cardiovascular risk factor, and dietary modulation via nutraceuticals represents a promising preventive strategy. Curcumin, a bioactive polyphenol from *Curcuma longa* L., exhibits pleiotropic biological activities; however, its capacity to reduce circulating Hcy remains uncertain.

**Objectives:**

To systematically evaluate the effect of curcumin supplementation on Hcy levels.

**Methods:**

Eight databases were searched from inception to November 2025 for RCTs investigating curcumin and Hcy outcomes. Risk of bias was assessed using Cochrane RoB 2.0. A random-effects model was applied.

**Results:**

Three randomized controlled trials (RCT) with a total of 110 subjects were included in this study. Due to physiologically implausible Hcy values in one study (Rezaei 2024), the primary meta-analysis was restricted to two RCTs with interpretable data (Ghaffari 2017 and Campbell 2019). The results of Meta-analysis showed that compared with the control group, the pooled standardized mean difference SMD = −2.08, 95% confidence interval was −4.67 to 0.51, P = 0.116, there was no statistically significant difference between the two groups. There was a high degree of heterogeneity among the included studies (I^2^ = 91.6%, P = 0.001). Risk of bias assessment (RoB 2.0) rated one study as low risk, one as some concerns, and one as high risk (selective reporting). Sensitivity analysis that included the study with implausible data (Rezaei 2024) gave a similar non-significant pooled estimate (SMD = −1.23, 95% CI −2.82 to 0.36, P = 0.13; I^2^ = 91.9%).

**Conclusion:**

Current RCT evidence does not support a statistically significant Hcy-lowering effect of curcumin. The overall certainty of evidence is very low due to small sample size, extreme heterogeneity, and risk of bias. Curcumin should not be recommended as a standalone intervention for lowering Hcy. Its potential role, if any, requires confirmation in well-powered trials using optimized, high-bioavailability formulations.

## 1 Introduction

Homocysteine (Hcy) is a sulfur-containing amino acid generated as an intermediate in methionine metabolism. Elevated plasma Hcy—clinically defined as hyperhomocysteinemia (HHcy)—has been recognized as an independent risk factor for a spectrum of chronic diseases. Extensive epidemiological evidence links HHcy with increased risk of cardiovascular and cerebrovascular events, including coronary artery disease and ischemic stroke (Clarke et al., 2011; Finkelstein and Martin, 2000). Beyond vascular sequelae, elevated Hcy is implicated in the pathogenesis of neurodegenerative disorders such as Alzheimer’s disease (Ortiz-Salguero et al., 2024; Bonetti et al., 2016), osteoporosis (Herrmann et al., 2007), and diabetic vascular complications (Cheng et al., 2015), primarily through induction of oxidative stress, endothelial dysfunction, and pro-inflammatory signaling (Smith and Refsum, 2021). Given the widespread prevalence and modifiable nature of elevated Hcy, identifying safe and effective dietary strategies to lower Hcy is of considerable public health importance.

Curcumin (1,7-bis(4-hydroxy-3-methoxyphenyl)-1,6-heptadiene-3,5-dione), the principal bioactive curcuminoid isolated from the rhizome of *Curcuma longa* L. (turmeric), has attracted substantial interest as a multitarget nutraceutical. Curcumin exerts anti-inflammatory, antioxidant, lipid-lowering, and vascular-protective activities through modulation of multiple signaling pathways, making it a candidate for adjunctive management of cardiometabolic disease (Gupta et al., 2013; Aggarwal and Harikumar, 2009). From a nutritional perspective, curcumin exemplifies the concept of “food-medicine homology” (Hewlings and Kalman, 2017), with a favorable safety profile that supports its use as a functional food ingredient. Mechanistically, curcumin may influence Hcy metabolism by activating folate-dependent one-carbon metabolic pathways, upregulating cystathionine β-synthase, and attenuating oxidative stress that impairs Hcy transsulfuration (Mondal et al., 2019). However, the evidence from clinical trials remains inconsistent, with some studies reporting significant Hcy reductions (Mondal et al., 2019) and others showing neutral or even contrary effects.

The inconsistency across trials may reflect heterogeneity in participant characteristics, curcumin dose and formulation, intervention duration, dietary co-interventions (e.g., folate and B-vitamin intake), and baseline Hcy status. To date, no comprehensive meta-analysis has systematically integrated available clinical evidence and quantitatively explored the moderating factors. The present study aimed to: (i) systematically synthesize RCT evidence on curcumin supplementation and Hcy levels and (ii) quantify the overall effect using meta-analytic methods.

## 2 Materials and methods

### 2.1 Study design and registration

This systematic review and meta-analysis was conducted in accordance with the Preferred Reporting Items for Systematic Reviews and Meta-Analyses (PRISMA) 2020 guidelines (Page et al., 2021; Muka et al., 2020). The protocol was prospectively registered in the PROSPERO international registry (CRD42024576272).

### 2.2 Search strategy

A systematic literature search was performed across eight electronic databases: PubMed, Embase, Cochrane Central Register of Controlled Trials, Web of Science, China National Knowledge Infrastructure (CNKI), Wanfang Data, VIP Chinese Sci-tech Journals Database, and China Biology Medicine disc (CBM). The search covered all records from database inception to November 2025 without language restriction. The search strategy combined MeSH terms and free-text keywords related to curcumin (e.g., “curcumin,” “diferuloylmethane,” “turmeric”) and homocysteine (e.g., “homocysteine,” “hyperhomocysteinemia,” “Hcy”). The full Embase search string is provided in the Supplementary Material.

### 2.3 Eligibility criteria

Studies were included if they met all of the following PICOS criteria (Table 1): (1) Population—adults aged ≥18 years, regardless of health status; (2) Intervention—curcumin or a curcumin-containing preparation as the primary experimental intervention, with clearly reported dose, route of administration, and duration; (3) Comparator—placebo or standard care, excluding co-interventions known to affect Hcy (e.g., folic acid, B vitamins); (4) Outcome—serum or plasma Hcy levels with extractable quantitative data (mean and standard deviation, pre- and post-intervention or between groups); (5) Study design—randomized controlled trial (RCT), regardless of blinding status.

**TABLE 1:** PICOS criteria for study inclusion and exclusion.

Parameter	Criteria
Population	Adults aged ≥18 years (healthy or with chronic conditions)
Intervention	Curcumin or curcumin-containing preparation as primary intervention (dose, route, and duration clearly reported)
Comparator	Placebo or standard care (excluding folate or B-vitamin co-interventions)
Outcome	Serum/plasma homocysteine levels (mean ± SD extractable at baseline and follow-up)
Study Design	Randomized controlled trial (RCT)

Studies were excluded if they were: non-clinical (animal or *in vitro*) investigations; reviews, meta-analyses, conference abstracts, or dissertations without a corresponding peer-reviewed journal publication; reports with incomplete or irreconcilable outcome data; or duplicate publications (the most complete version was retained).

### 2.4 Study selection and data extraction

All identified records were imported into NoteExpress for duplicate removal. Two independent reviewers screened titles and abstracts, followed by full-text assessment of potentially eligible records. Discrepancies were resolved through discussion; a third senior investigator adjudicated unresolved disagreements. Data were extracted using a standardized, pre-piloted form capturing: first author, publication year, country, study design, population characteristics (sample size, age, sex distribution, health status), intervention details (curcumin dose, formulation, duration, control condition), and outcome data (mean ± SD of Hcy at baseline and follow-up, or change scores).

### 2.5 Risk of bias assessment

The methodological quality of included RCTs was evaluated independently by two reviewers using the Cochrane Risk of Bias tool version 2.0 (RoB 2.0) (Sterne et al., 2019; Cumpston et al., 2019), which assesses bias across five domains: (1) randomization process; (2) deviations from intended interventions; (3) missing outcome data; (4) measurement of the outcome; and (5) selection of the reported result. Each domain was rated as low risk, some concerns, or high risk. Overall bias judgments were derived from domain-level ratings.

### 2.6 Statistical analysis

All analyses were performed in Stata version 17.0 (StataCorp, College Station, TX, United States) (StataCorp. Stata Statistical Software, 2021). Because Hcy is a continuous outcome, the effect size was expressed as the standardized mean difference (SMD) with 95% confidence intervals (CIs). Because baseline Hcy levels varied substantially across included studies (range: 14.9–188 μmol/L), the standardized mean difference (SMD) was used in preference to the weighted mean difference to account for differences in measurement scales and to improve comparability. Statistical heterogeneity was quantified using the I^2^ statistic and Cochran’s Q test. Given the clinical and methodological diversity of included trials, a DerSimonian–Laird random-effects model was applied *a priori* (Cumpston et al., 2019). Results were visualized as forest plots. Sensitivity analysis was conducted by sequentially omitting each study to evaluate the robustness of the pooled estimate. Publication bias was assessed visually using funnel plots; Egger’s regression test and Meta regression analysis was not performed because fewer than ten studies were available (Sterne et al., 2011).

## 3 Results

### 3.1 Study selection

**FIGURE 1:**
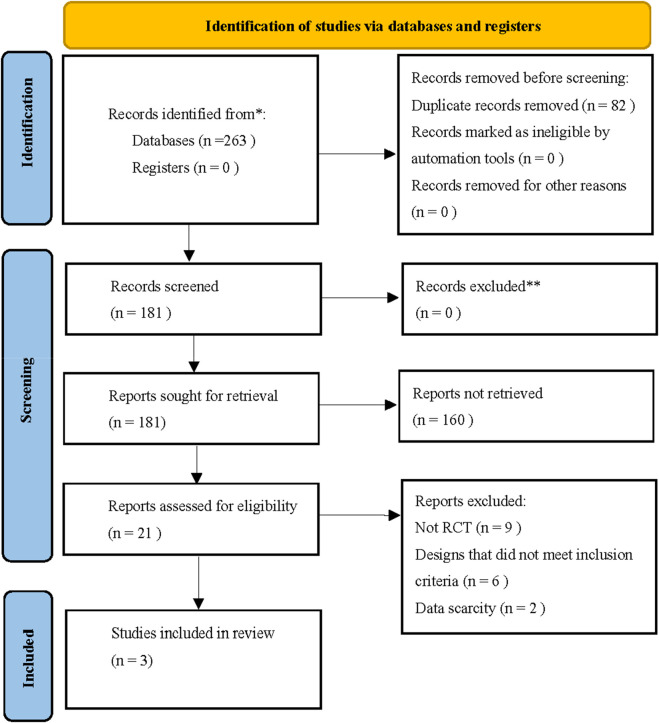
Flow diagram of study selection.

The database search identified 263 records. After removing 82 duplicates with NoteExpress, 181 records were screened by title and abstract; 160 were excluded as clearly irrelevant. Full-text review of the remaining 21 records led to the exclusion of 16 studies (non-RCT, n = 9; design not meeting inclusion criteria, n = 6; insufficient data, n = 2; other, n = 1). Three RCTs (Rezaei et al., 2024; Ghaffari et al., 2017; Campbell et al., 2019) were ultimately included in the quantitative synthesis (Figure 1).

### 3.2 Study characteristics

The three included trials enrolled 110 participants from Iran and the United States. Study populations varied considerably: overweight or obese patients with coronary slow flow phenomenon (CSFP; Rezaei et al. (2024)), patients with non-alcoholic fatty liver disease (NAFLD; Ghaffari et al., 2017), young obese men (Campbell et al. (2019)). Intervention duration was 12 weeks; daily curcumin dose varied widely, from 0.08 g to 3.0 g. Baseline Hcy levels differed substantially across studies. Detailed characteristics are presented in Table 2.

**TABLE 2:** Characteristics of included studies.

References	Country	Design	Population	Mean age (y)	n (total)	Duration	Daily dose (g)	Control	Baseline Hcy (μmol/L)	Endpoint Hcy (μmol/L)
Rezaei et al. (2024)	Iran	Parallel	Overweight/obese patients with CSFP	54.3	42	12 weeks	0.08	Placebo	188.0 ± 290.1^a^	211.4 ± 335.0^a^
Ghaffari et al. (2017)	Iran	Parallel	Patients with NAFLD	20–60	46	12 weeks	3.00	Placebo	14.91 ± 6.69	11.46 ± 3.56
Campbell et al. (2019)	United States	Parallel	Young obese men	18–35	22	12 weeks	0.50	Placebo	90.4 ± 16.9^b^	63.8 ± 7.5^b^

^a^ Data are physiologically implausible (mean ± SD). The authors have been contacted for verification; this study is included only in sensitivity analyses.

^b^ Original unit was μg/mL; converted to μmol/L (×7.4). Original values: curcumin group 12.22→8.62 μg/mL, placebo group 9.45→11.84 μg/mL.

### 3.3 Risk of bias

Risk of bias assessment is summarized in Table 3. One study (Ghaffari et al., 2017) was low risk, one study (Rezaei et al., 2024) was some concern, and one study (Campbell 2019) was high risk. The results of subdomain evaluation showed that all included studies were low-risk in the four core areas of randomization process, deviation from the established intervention, missing outcome data and outcome measurement. Ghaffari et al., 2017 reported outcome indicators were consistent with the prespecified protocol and rated as low risk. Although Rezaei et al., 2024 has been registered in the Clinical Trials Registration Platform of Iran, the Supplementary Material (medication record form) are not fully included in the main article, so Rezaei et al., 2024 is rated as some concerns. Campbell et al., 2019 did not provide clinical trial registration number or pre-registration plan, and selective reporting bias could not be ruled out and was rated as high risk. The detailed risk-of-bias assessments for each domain are shown in Table 3.

**TABLE 3:** Risk-of-bias assessment (cochrane RoB 2.0).

References	Randomization process	Deviations from intended interventions	Missing outcome data	Measurement of the outcome	Selection of the reported result	Overall risk
Rezaei et al. (2024)	Low	Low	Low	Low	Some concerns^b^	Some concerns
Ghaffari et al. (2017)	Low	Low	Low	Low	Low	Low
Campbell et al. (2019)	Low	Low	Low	Low	High^a^	High

^a^ No clinical trial registration number or pre-registered protocol was provided; selective reporting cannot be ruled out.

^b^ Registered in the Iranian Registry of Clinical Trials, but Supplementary Material (medication use table) was not fully included in the main article.

### 3.4 Meta-analysis

**FIGURE 2:**
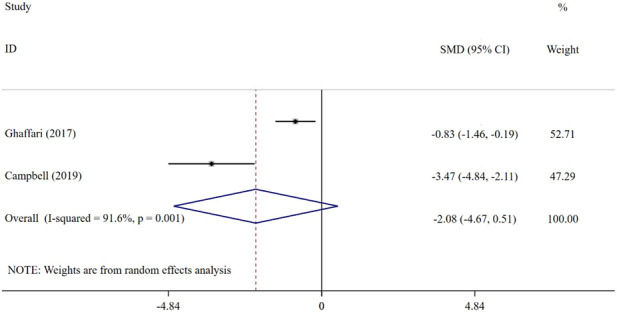
Forest plot of randomized controlled trials investigating the effects of curcumin supplementation on homocysteine levels.

Among the three included RCTs, one study (Rezaei et al., 2024) reported Hcy values that were physiologically implausible (mean 188.0 μmol/L with a standard deviation of 290.1 μmol/L, coefficient of variation >150%). Therefore, the primary meta-analysis was conducted on the two studies with interpretable data: Ghaffari et al., 2017 (NAFLD patients, turmeric powder 3 g/day) and Campbell et al., 2019 (young obese men, enhanced-bioavailability curcumin 500 mg/day). Pooled analysis was performed using the DerSimonian-Laird random-effects model. The results revealed that the pooled standardized mean difference (SMD) in Hcy levels between the curcumin supplementation group and the control group was −2.08 (95% confidence interval: −4.67 to 0.51, P = 0.116), indicating that curcumin supplementation had no significant effect on Hcy levels. Substantial heterogeneity was detected across the included studies (I^2^ = 91.6%, P = 0.001) (Figure 2).

### 3.5 Publication bias

**FIGURE 3:**
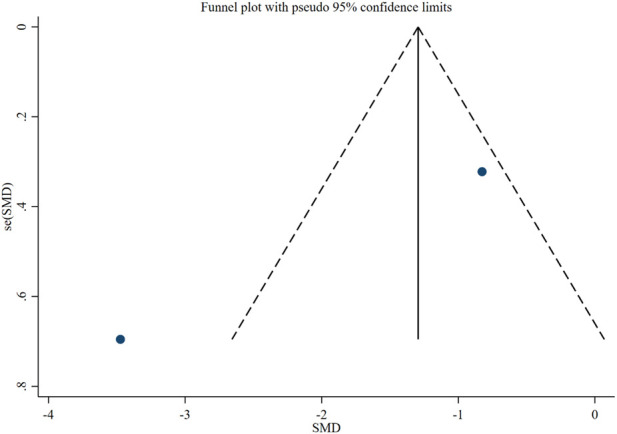
Funnel plot of randomized controlled trials investigating the effects of curcumin supplementation on homocysteine levels. (Note: due to fewer than 10 studies, Egger’s test was not performed).

The funnel plot did not display the expected symmetrical inverted-funnel pattern, suggesting a potential risk of publication bias (Figure 3). Formal Egger’s test was not conducted given the small number of included studies (n < 10), as recommended in current reporting guidelines (Sterne et al., 2011).

### 3.6 Sensitivity analysis

To assess the robustness of the primary findings and explore sources of heterogeneity, several sensitivity analyses were performed.

Leave-one-out analysis: Sequentially omitting each study from the three-study set showed that the direction of the pooled effect did not change, but the confidence intervals varied. When Rezaei et al., 2024 was omitted, the pooled SMD (Ghaffari et al., 2017 + Campbell et al., 2019) was −2.08 (95% CI: −4.67 to 0.51, P = 0.116), as reported in the primary analysis. Omission of Ghaffari et al., 2017 or Campbell et al., 2019 resulted in pooled estimates with wide confidence intervals crossing zero (data not shown).

Inclusion of the study with implausible data: When Rezaei et al., 2024 (with its original reported values) was included in the meta-analysis together with the other two studies, the pooled SMD was −1.23 (95% CI: −2.82 to 0.36, P = 0.13), with I^2^ = 91.9% (P < 0.001). This result remained non-significant and highly heterogeneous, similar to the primary analysis.

Exclusion of the high-risk-of-bias study: After excluding Campbell 2019 (rated high risk due to lack of pre-registration), the pooled SMD of the remaining two studies (Ghaffari et al., 2017 and Rezaei et al., 2024) was −0.30 (95% CI: −1.33 to 0.73, P = 0.57), with I^2^ = 81.9% (P = 0.019). The non-significant finding persisted.

**FIGURE 4:**
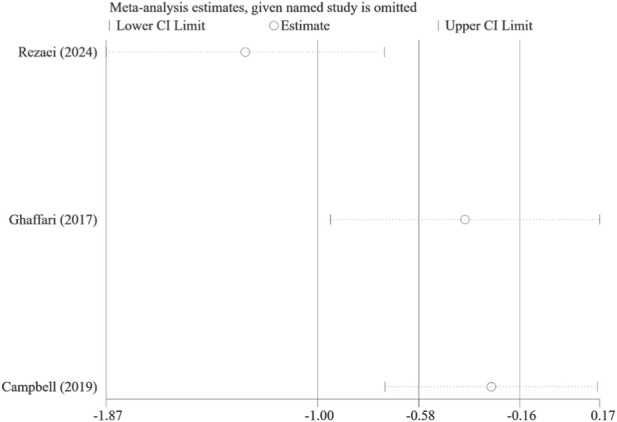
Sensitivity analysis.

These sensitivity analyses indicate that the overall conclusion—no statistically significant Hcy-lowering effect of curcumin—is robust to different analytical decisions, although the high heterogeneity remains unexplained (Figure 4).

### 3.7 Grade certainty of evidence assessment

Using the GRADE (Grading of Recommendations Assessment, Development and Evaluation) approach, the overall certainty of evidence for the effect of curcumin on Hcy levels was rated as very low.

This rating was based on: (1) serious risk of bias (one included study had high risk of bias due to lack of pre-registration; another raised some concerns); (2) serious inconsistency (substantial heterogeneity, I^2^ > 90%, which could not be explained by sensitivity analyses); (3) serious imprecision (the 95% confidence interval of the pooled effect was wide and crossed the null, and the total sample size was small, n = 110); and (4) indirectness (the studies used different curcumin formulations—ordinary powder, enhanced-bioavailability, nano-curcumin—and enrolled different populations, limiting direct applicability to a general healthy population). No serious publication bias was judged, but formal testing was not possible due to the small number of studies. Therefore, future research is very likely to change the estimate of effect.

## 4 Discussion

To our knowledge, this is the first systematic review and meta-analysis to comprehensively evaluate the effect of curcumin supplementation on circulating Hcy levels. Among the three included RCTs, one study (Rezaei et al., 2024) reported Hcy values that were physiologically implausible (mean 188.0 μmol/L with a standard deviation of 290.1 μmol/L, coefficient of variation >150%). Therefore, the primary meta-analysis was conducted on the two studies with interpretable data: Ghaffari et al., 2017 (NAFLD patients, turmeric powder 3 g/day) and Campbell 2019 (young obese men, enhanced-bioavailability curcumin 500 mg/day) (SMD = −2.08; 95% CI: −4.67 to 0.51, P = 0.116). However, this null overall result must be interpreted in the context of substantial between-trial heterogeneity (I^2^ = 91.6%, P = 0.001) and small evidence base, both of which substantially limit the precision and generalizability of the pooled estimate.

The high heterogeneity observed in our analysis is not surprising given the substantial variation in trial characteristics. First, baseline Hcy levels differed across study populations. Since curcumin’s effect on one-carbon metabolism may be more pronounced in individuals with pre-existing metabolic dysregulation or micronutrient insufficiency, studies enrolling populations with higher baseline Hcy may be more likely to observe a meaningful response. Second, the curcumin formulations used across studies differed markedly—conventional curcumin (with low oral bioavailability estimated at <1%) was used in some trials, while enhanced-bioavailability formulations (nano-curcumin, phospholipid complexes) were employed in others. Given that the pharmacokinetic profile of curcumin fundamentally determines tissue exposure and downstream biological effects, formulation differences may have contributed substantially to heterogeneity. Third, none of the included studies reported systematic control of dietary folate or B-vitamin intake—key cofactors in Hcy remethylation—which represents a major potential confounding variable. Furthermore, leave-one-out sensitivity analysis confirmed that the study by Campbell et al. (2019) was the primary source of heterogeneity. Assessed by the Cochrane Risk-of-Bias Tool 2.0 (RoB 2.0), this trial had a high overall risk of bias mainly attributed to selective reporting, which further exacerbated inter-study heterogeneity.

Although we could not perform meta-regression due to the limited number of included studies, existing mechanistic evidence indicates that curcumin’s Hcy-lowering effect is dose- and formulation-dependent. Curcumin is proposed to lower Hcy by activating cystathionine β-synthase (CBS) in the transsulfuration pathway and by reducing oxidative stress-driven inhibition of methylenetetrahydrofolate reductase (MTHFR), both of which are dose-sensitive processes. A certain pharmacological threshold of curcumin exposure is required to exert stable effects, which may not have been consistently achieved in the included trials. This finding has important implications for future trial design: investigators should prioritize dosing regimens based on pharmacokinetic modeling rather than empirical selection.

From a nutritional science perspective, these findings contribute to the growing understanding of curcumin as a functional food bioactive with pleiotropic cardiometabolic effects. Curcumin’s capacity to modulate lipid metabolism, vascular inflammation, and endothelial function through multiple molecular targets has been well-documented [8,21,22]. The present analysis suggests that Hcy regulation may represent an additional—but dose-contingent and formulation-dependent—mechanism through which curcumin confers cardiovascular benefit. These findings support continued investigation of curcumin as part of multitarget dietary strategies for cardiovascular risk reduction, even in the absence of a robust isolated Hcy-lowering effect.

Several limitations of the present study warrant acknowledgment. The small number of included RCTs (n = 3, N = 185) severely restricts statistical power and the reliability of subgroup analyses. The substantial heterogeneity observed precluded meaningful subgroup analyses by disease population or formulation type. According to RoB 2.0 assessment, all trials were rated as low risk of bias in core domains including randomisation process, deviations from intended interventions, missing outcome data and outcome measurement; nevertheless, several studies had concerns or high risk regarding selection of reported results. Additionally, the potential for publication bias cannot be excluded. Although formal testing for publication bias was not possible due to the small number of studies (n < 10), the observed funnel plot asymmetry (Figure 3) warrants consideration. Because the pooled effect estimate was not statistically significant and the 95% confidence interval was wide, the presence of publication bias—which typically favors positive or significant results—is unlikely to reverse the null conclusion. However, the asymmetry may indicate that smaller studies with null or negative results are missing from the literature, which would further weaken the evidence for any beneficial effect. Thus, while our main conclusion remains robust, readers should be aware of the potential for unpublished null studies. Finally, none of the studies reported long-term follow-up or systematically monitored curcumin-drug interactions, which are important considerations for clinical translation.

Future research should address these gaps through: (1) well-powered, double-blind RCTs using standardized, high-bioavailability curcumin formulations (e.g., liposomal, phospholipid complexes, or nanoparticle-based preparations) at doses ≥0.5 g/day; (2) strict dietary control of folate and B-vitamin intake, and measurement of key one-carbon metabolites (e.g., methylmalonic acid, B12, folate) at baseline and follow-up; (3) stratification by baseline Hcy status and metabolic phenotype to identify populations most likely to benefit; and (4) integration of mechanistic biomarkers (e.g., CBS activity, MTHFR genotyping) to elucidate the biological basis of curcumin’s effect on Hcy metabolism.

## 5 Conclusion

This systematic review and meta-analysis found no statistically significant effect of curcumin supplementation on circulating Hcy levels based on the two available parallel-group RCTs with interpretable data (SMD = −2.08; 95% CI: −4.67 to 0.51; P = 0.116). However, the overall certainty of evidence was rated as very low using the GRADE approach (Section 3.7). This rating reflects several critical limitations: the very small total sample size (N = 110), extreme and unexplained between-study heterogeneity (I^2^ = 91.6%), and a high risk of bias (selective reporting) in one of the two primary studies. Because of these severe limitations, the true effect of curcumin on Hcy may be substantially different from the present pooled estimate, and no definitive conclusion can be drawn.

Therefore, curcumin should not currently be recommended as a standalone intervention for lowering Hcy in any population. Its potential benefit—if any—remains speculative and requires confirmation in adequately powered, well-designed randomized controlled trials using standardized, high-bioavailability formulations and rigorous control of dietary folate and B-vitamin intake. Any clinical or nutritional recommendations must await such evidence.

## Data Availability

The original contributions presented in the study are included in the article/Supplementary Material, further inquiries can be directed to the corresponding authors.

## References

[B1] Aggarwal B. B. Harikumar K. B. (2009). Potential therapeutic effects of curcumin, the anti-inflammatory agent, against neurodegenerative, cardiovascular, pulmonary, metabolic, autoimmune and neoplastic diseases. Int. J. Biochem. Cell Biol. 41 (1), 40–59. 10.1016/j.biocel.2008.06.010 18662800 PMC2637808

[B2] Bonetti F. Brombo G. Zuliani G. (2016). The relationship between hyperhomocysteinemia and neurodegeneration. Neurodegener. Dis. Manag. 6 (2), 133–145. 10.2217/nmt-2015-0008 27033101

[B3] Campbell M. S. Ouyang A. I M K. Charnigo R. J. Westgate P. M. Fleenor B. S. (2019). Influence of enhanced bioavailable curcumin on obesity-associated cardiovascular disease risk factors and arterial function: a double-blinded, randomized, controlled trial. Nutrition 62, 135–139. 10.1016/j.nut.2019.01.002 30889454

[B4] Cheng Z. Jiang X. Pansuria M. Fang P. Mai J. Mallilankaraman K. (2015). Hyperhomocysteinemia and hyperglycemia induce and potentiate endothelial dysfunction via μ-calpain activation. Diabetes 64 (3), 947–959. 10.2337/db14-0784 25352635 PMC4338586

[B5] Clarke R. Halsey J. Bennett D. Lewington S. (2011). Homocysteine and vascular disease: review of published results of the homocysteine-lowering trials. J. Inherit. Metab. Dis. 34 (1), 83–91. 10.1007/s10545-010-9235-y 21069462

[B6] Cumpston M. Li T. Page M. J. Chandler J. Welch V. A. Higgins J. P. (2019). Updated guidance for trusted systematic reviews: a new edition of the cochrane handbook for systematic reviews of interventions. Cochrane Database Syst. Rev. 10, ED000142. 10.1002/14651858.ED000142 31643080 PMC10284251

[B7] Finkelstein J. D. Martin J. J. (2000). Homocysteine. Int. J. Biochem. Cell Biol. 32 (4), 385–389. 10.1016/s1357-2725(99)00138-7 10762063

[B8] Ghaffari A. Rafraf M. Navekar R. Sepehri B. Asghari-Jafarabadi M. Ghavami S. M. (2017). Effects of turmeric on homocysteine and fetuin-A in patients with non-alcoholic fatty liver disease: a randomized double-blind placebo-controlled study. Iran. Red. Crescent Med. J. 19 (4), e43193. 10.5812/ircmj.43193

[B9] Gupta S. C. Sung B. Kim J. H. Prasad S. Li S. Aggarwal B. B. (2013). Multitargeting by turmeric, the golden spice: from kitchen to clinic. Mol. Nutr. Food Res. 57 (9), 1510–1528. 10.1002/mnfr.201100741 22887802

[B10] Herrmann M. Peter Schmidt J. Umanskaya N. Wagner A. Taban-Shomal O. Widmann T. (2007). The role of hyperhomocysteinemia as well as folate, vitamin B6 and B12 deficiencies in osteoporosis: a systematic review. Clin. Chem. Lab. Med. 45 (12), 1621–1632. 10.1515/CCLM.2007.362 18067447

[B11] Hewlings S. J. Kalman D. S. (2017). Curcumin: a review of its effects on human health. Foods 6 (10), 92. 10.3390/foods6100092 29065496 PMC5664031

[B12] Mondal N. K. Behera J. Kelly K. E. George A. K. Tyagi P. K. Tyagi N. (2019). Tetrahydrocurcumin epigenetically mitigates mitochondrial dysfunction in brain vasculature during ischemic stroke. Neurochem. Int. 122, 120–138. 10.1016/j.neuint.2018.11.015 30472160 PMC6666268

[B13] Muka T. Glisic M. Milic J. Verhoog S. Bohlius J. Bramer W. (2020). A 24-step guide on how to design, conduct, and successfully publish a systematic review and meta-analysis in medical research. Eur. J. Epidemiol. 35 (1), 49–60. 10.1007/s10654-019-00576-5 31720912

[B14] Ortiz-Salguero C. Romero-Bernal M. González-Díaz Á. Doush E. S. Del Río C. Echevarría M. (2024). Hyperhomocysteinemia: underlying links to stroke and hydrocephalus, with a focus on polyphenol-based therapeutic approaches. Nutrients 17 (1), 40. 10.3390/nu17010040 39796474 PMC11722995

[B15] Page M. J. McKenzie J. E. Bossuyt P. M. Boutron I. Hoffmann T. C. Mulrow C. D. (2021). The PRISMA 2020 statement: an updated guideline for reporting systematic reviews. BMJ 372, n71. 10.1136/bmj.n71 33782057 PMC8005924

[B16] Rezaei M. Soltani M. Alipoor E. Rezayat S. M. Vasheghani-Farahani A. Yaseri M. (2024). Effect of nano-curcumin supplementation on angina status and cardiovascular risk factors in overweight or Obese patients with coronary slow flow phenomenon: a randomized double-blind placebo-controlled trial. BMC Nutr. 10 (1), 73. 10.1186/s40795-024-00877-3 38741194 PMC11089698

[B17] Smith A. D. Refsum H. (2021). Homocysteine—From disease biomarker to disease prevention. J. Intern. Med. 290 (4), 826–854. 10.1111/joim.13279 33660358

[B18] Statacorp. Stata Statistical Software: Release 17. College Station, TX: StataCorp LLC; (2021).

[B19] Sterne J. A. C. Sutton A. J. Ioannidis J. P. A. Terrin N. Jones D. R. Lau J. (2011). Recommendations for examining and interpreting funnel plot asymmetry in meta-analyses of randomised controlled trials. BMJ 343, d4002. 10.1136/bmj.d4002 21784880

[B20] Sterne J. A. C. Savović J. Page M. J. Elbers R. G. Blencowe N. S. Boutron I. (2019). RoB 2: a revised tool for assessing risk of bias in randomised trials. BMJ 366, l4898. 10.1136/bmj.l4898 31462531

