# Supplementary material for: Effects of curcumin supplementation on homocysteine levels: a systematic review and meta-analysis of randomized controlled trials
Source: Front Pharmacol. 2026 Jul 16;17:1864151. doi: 10.3389/fphar.2026.1864151 (PMC13422230; doi:10.3389/fphar.2026.1864151)
Supplement: Supplementary file 1 [file Table1.docx]

**Effects of Curcumin Supplementation on Homocysteine Levels: A Systematic Review and Meta-Analysis of Randomized Controlled Trials**

Hong Cao ^1,2,3,4,5#^, Wenrui Ning ^2#^, Feng Zhang ^1,2,3,4^, Xin Zhang ^3^, Jiai Yan ^1,2,3^,

Yingyu Wang ^1,2,3^, Jing Sun ^1,2,3^, Yiran Liu ^1,2,3^, Dan Li ^1,2,3*^, Ju Yang ^1,2,3*^

1 Nutrition Department (Center for Clinical Evaluation of Functional Foods), Affiliated Hospital of Jiangnan University, Wuxi，214122, China;

2 School of Biotechnology, Jiangnan University, Wuxi, 214122, China;

3 Wuxi Medical College, Jiangnan University, Wuxi, 214122, China;

4 Jiangsu Jicui Future Food Technology Research Institute Co., Ltd., Yixing, 214213, China;

5 Endocrine Department, Jiangnan University Affiliated Hospital, Wuxi, 214122, China.

# These authors contributed equally. * Corresponding authors.

*Corresponding authors at: No. 1000 Hefeng Road, Binhu District, Wuxi, China.

E-mail addresses: yangju0909@163.com (Ju Yang), danliyyh@jiangnan.edu.cn (Dan Li).

**Supplementary Table1 Search strategies:**

|  | **PubMed** |
| --- | --- |
| **#1** | **homocysteine OR hyperhomocysteinemia OR 2-amino-4-mercaptobutyric acid OR 2 amino 4 mercaptobutyric acid OR Homocysteine, L-Isomer OR Homocysteine, L Isomer OR L-Isomer Homocysteine OR Hcy** |
| **#2** | **curcumin OR 1,6-Heptadiene-3,5-dione, 1,7-bis(4-hydroxy-3-methoxyphenyl)-, (E,E)- OR Diferuloylmethane** |
| **#3** | **#1 AND #2** |

|  | **Embase** |
| --- | --- |
| **#1** | **"homocysteine" OR "hyperhomocysteinemia" OR "2-amino-4-mercaptobutyric acid" OR "2 amino 4 mercaptobutyric acid" OR "Homocysteine, L-Isomer" OR "Homocysteine, L Isomer" OR "L-Isomer Homocysteine" OR "Hcy"** |
| **#2** | **"curcumin" OR "1,6-Heptadiene-3,5-dione", "1,7-bis(4-hydroxy-3-methoxyphenyl)-, (E,E)-" OR "Diferuloylmethane"** |
| **#3** | **#1 AND #2** |

|  | **Cochrane Library** |
| --- | --- |
| **#1** | **MeSH descriptor: [Homocysteine] explode all trees** |
| **#2** | **(homocysteine):ti,ab,kw OR(hyperhomocysteinemia):ti,ab,kw OR(2 amino 4 mercaptobutyric acid):ti,ab.kw OR(Homocysteine, LIsomer):ti,ab,kw OR(L-Isomer Homocysteine):ti,ab,kw OR(Hcy):ti,ab,kw** |
| **#3** | **#1 OR #2** |
| **#4** | **MeSH descriptor:[Curcumin] explode all trees** |
| **#5** | **(Curcumin):ti,ab,kw OR(Curcumin Phytosome)ti,ab,kw OR(Phytosome Curcumin):ti,ab,kw OR(1 6 Heptadiene 3 5 dione 17 bis(4 hydroxy3methoxypheny)(E EJ):ti,ab,kw OR(Oiferuloyimethane)ti,ab,kw OR(Turmeric Yellow):ti.ab,kw OR(Yellow Turmeric):ti,ab,kw OR(Mervia):ti,ab,kw** |
| **#6** | **#4 OR #5** |
| **#7** | **#3 AND #6** |

|  | **Web of Science** |
| --- | --- |
| **#1** | **homocysteine OR hyperhomocysteinemia OR "2-amino-4-mercaptobutyric acid" OR "2 amino 4 mercaptobutyric acid" OR "Homocysteine, L-Isomer" OR "Homocysteine, L Isomer" OR "L-Isomer Homocysteine" OR Hcy** |
| **#2** | **curcumin OR "1,6-Heptadiene-3,5-dione, 1,7-bis(4-hydroxy-3-methoxyphenyl)-, (E,E)-" OR Diferuloylmethane** |
| **#3** | **#1 AND #2** |

|  | **Wanfang Data Knowledge Service Platform** |
| --- | --- |
| **#1** | **(主题:(同型半胱氨酸) or 题名或关键词:(同型半胱氨酸 or 血同型半胱氨酸 or血清同型半胱氨酸or 高同型半胱氨酸 or高同型半胱氨酸血症or总同型半胱氨酸or高半胱氨酸or异构体同型半胱氨酸or2-氨基-4-巯基丁酸)) and(主题:(姜黄素)题名或关键词:(姜黄素or1,7-双(4-羟基-3-甲氧基苯基)-1,6-二烯-**  **3,5-庚二酮)** |

|  | **China National Knowledge Infrastructure (CNKI)** |
| --- | --- |
| **#1** | **同型半胱氨酸 OR 血同型半胱氨酸 OR 血清同型半胱氨酸 OR 高同型半胱氨酸 OR 高同型半胱氨酸血症 OR 总同型半胱氨酸 OR 高半胱氨酸 OR 异构体同型半胱氨酸 OR "2-氨基-4-巯基丁酸** |
| **#2** | **姜黄素 OR "1,7-双(4-羟基-3-甲氧基苯基)-1,6-二烯-3,5-庚二酮** |
| **#3** | **#1 AND #2** |

|  | **VIP Chinese Sci-tech Journals Database (VIP)** |
| --- | --- |
| **#1** | **同型半胱氨酸 OR 血同型半胱氨酸 OR 血清同型半胱氨酸 OR 高同型半胱氨酸 OR 高同型半胱氨酸血症 OR 总同型半胱氨酸 OR 高半胱氨酸 OR 异构体同型半胱氨酸 OR "2-氨基-4-巯基丁酸** |
| **#2** | **姜黄素 OR "1,7-双(4-羟基-3-甲氧基苯基)-1,6-二烯-3,5-庚二酮** |
| **#3** | **#1 AND #2** |

|  | **China Biology Medicine disc (CBM)** |
| --- | --- |
| **#1** | **"高半胱氨酸"[不加权:扩展]** |
| **#2** | **"同型半胱氨酸"[常用字段:智能]OR"血同型半胱氨酸[常用字段:智能]OR"血清同型半胱氨酸"[常用字段:智能]OR"高同型半胱氨酸'[常用字段:智能]OR"高同型半胱氨酸血症"[常用字段:智能]OR"总同型半胱氨酸"[常用字段:智能]OR"高半胱氨酸"[常用字段:智能]OR"异构体同型半胱氨酸"[常用字段:智能]OR"2-氨基-4-巯基丁酸"[常用字段:智能]** |
| **#3** | **#1 OR #2** |
| **#4** | **"姜黄素"[不加权:扩展]** |
| **#5** | **"姜黄素"[常用字段:智能]OR1,7-双(4-羟基-3-甲氧基苯基)-1,6-二烯-3,5-庚二酮**  **4"姜黄素"[不加权:扩展]** |
| **#6** | **#4 OR #5** |
| **#7** | **#3 AND #6** |

Supplementary Table 2 Original data unit conversion

| Reference | Original data | Converted data(µmol/L) |
| --- | --- | --- |
| Rezaei  2024 | 188.0 ± 290.1mcmol/L | 188.0 ± 290.1 |
| Ghaffari  2017 | 14.91 ±6.69µmol/L | 14.91 ±6.69 |
| Campbell  2019 | 18.46 ± 8.45µg/mL | 90.4 ± 16.9 |
